# Diabetes type 2: relationships between lysosomal exocytosis of circulating normal-sized platelets and *in vitro* ɑ-thrombin-evoked platelet responses

**DOI:** 10.1080/07853890.2023.2171108

**Published:** 2023-03-16

**Authors:** Maria Edvardsson, Magnus Oweling, Petter Järemo

**Affiliations:** a Local Healthcare Center, Finspång, Sweden; b Department of Biomedical and Clinical Sciences, Linköping University, Linköping, Sweden; c Department of Internal Medicine, Vrinnevi Hospital, Norrköping, Sweden

**Keywords:** Annexin V, α_IIb_β_3_ activity, lysosomal-associated membrane protein 1, platelets, platelet reactivity

## Abstract

**Background/objective:**

Type 2 diabetes is a major risk factor for atherosclerotic disease. It is well agreed that the reactivity of diabetic platelets is increased but how platelet reactivity regulates is unknown. In our laboratory, density separated platelets have been investigated extensively and high- and low-density platelets circulate in an activated state. The density distribution of circulating platelets is altered in diabetes type 2 as well. We hypothesize that such platelets modify whole blood (WB) *in vitro* α-thrombin-evoked (10 μM/mL) activity in type 2 diabetes. Thus, the study aims to identify features of circulating normal-sized density subpopulations affecting whole blood (WB) platelet reactivity in type 2 diabetes.

**Patients and methods:**

Patients with type 2 diabetes (*n* = 16) were enrolled. Their normal-sized platelets were divided into density subfractions (*n* = 16) using continuous polyvinylpyrrolidone-coated silica (Percoll™) gradients (density span, 1.090–1.040 kg/L) containing prostaglandin E_1_. The proportions (%) of such density-separated platelets expressing lysosomal-associated membrane protein 1 (LAMP-1) were analyzed using a flow cytometer. Further, determinations of WB ɑ-thrombin-evoked (10 U/mL) surface LAMP-1 (an assessment of lysosomal release), the fibrinogen (α_IIb_β_3)_ receptor activity, annexin V (binds to exposed membrane phosphatidylserine), and mitochondrial transmembrane potentials (an estimate of organelle integrity) were performed. Surface LAMP-1 expressions of individual normal-sized platelet density subpopulations were stratified into equal-sized groups (*n* = 2) depending on reactivity, as judged from the ɑ-thrombin-induced WB activity markers.

**Results:**

With some exceptions, the proportion of normal-sized circulating platelets showing spontaneous LAMP-1 was strongly associated with WB ɑ-thrombin-evoked (10 U/mL) surface LAMP-1 and α_IIb_β_3_ receptor activity. LAMP-1-expressing normal-sized platelets also displayed inverse associations with WB ɑ-thrombin-induced surface annexin V and mitochondrial damage, which are features of procoagulant platelets.

**Conclusions:**

From the current descriptive work only involving type 2 diabetes, it is impossible to judge whether the findings are features of the disease or if they occur in healthy individuals as well. However, the study describes LAMP-1 expressing subpopulations of circulating normal-sized platelets that associate with WB α-thrombin (10 U/mL) responses *in vitro*. Increased proportions of such platelets induced lysosomal release and α_IIb_β_3_ receptor activity, whereas lower proportions promoted WB agonist-induced procoagulant platelet creation. It is to hypothesize that the new described regulatory mechanism could in the future offer a possibility to influence platelet behavior in type 2 diabetes.

**Key messages**
- Lysosomal exocytosis of circulating platelets influences reactivity, as determined by agonist-induced platelet reactions *in vitro*
- Thus, the low release of lysosomes by normal-sized platelets *in vivo* increases agonist-evoked procoagulant platelet production.
- Higher lysosomal exocytosis of circulating normal-sized platelets promotes platelet aggregation and secretion.

## Introduction

Platelets were deemed to be either resting or activated, and when activated, they were assumed to perform similar tasks. Recently, the binary view has been abandoned, and the concept that platelet functions are unevenly distributed has emerged [1,2]. Thus, if activated, some platelets discharge lysosomal constituencies, as evidenced by lysosomal-associated membrane protein 1 (LAMP-1), whereas others activate fibrinogen receptors (α_IIb_β_3_), thereby accomplishing aggregation. Furthermore, during activation, other platelets generate procoagulant corpuscles exposing phosphatidylserine (PS), as estimated from elevated surface annexin V levels, and increased mitochondrial injury [3–5]. PS enables the accumulation of surface prothrombinase complexes and accelerates platelet thrombin generation [6]. The mechanisms that steer the activation routes remain unknown. Recently, interest in platelet heterogeneity has increased, and the diversity of non-activated *in vitro* density-separated platelets has been studied by us [7–9]. Platelet density follows a Gaussian dispersion and varies within the range of 1.090–1.040 kg/L [10–12]. High-density platelets contain more granules (both ɑ- and dense) and mitochondria [10–12] and judging from fibrinogen expression, high- and low-density corpuscles circulate, showing a higher activation than the bulk of platelets [8,9].

Procoagulant platelets are clinically important [13,14] and many human diseases alter platelet diversity. Examples include tiny subfractions of larger platelets that forecast prostatic cancer relapse [15] and subpopulations of circulating Alzheimer platelets expressing less membrane-bound fibrinogen [16]. Furthermore, platelet density increases in conjunction with acute myocardial infarctions [17] and is then inversely linked to inflammatory reactions [18]. Moreover, high, and low platelet densities reflect inflammatory bowel disease activity [19] and preeclampsia severity [20], respectively. Type 2 diabetes enhances atherothrombotic risk and diabetic platelets demonstrate a higher baseline activity [21,22]. They also show increased activity both at baseline and in response to agonists such as ADP and α- thrombin [21–24]. Thus, impaired aspirin efficacy has been reported in conjunction with type 2 diabetes [25].

Multicolor flow cytometry is a powerful tool in platelet research. It can be used for low-platelet-count samples and is thus suitable for analyzing platelet subpopulations. The device separates corpuscles according to their relative size [4,26] and permits concurrent analysis of many activation markers. The current basic science study used this technique [4] and examined type 2 diabetes, i.e. a prothrombotic condition. The relationship between the activity of density-separated platelet subpopulations and whole blood (WB) ɑ-thrombin induced (10 U/mL) lysosomal release, α_IIb_β_3_ receptor activity, and procoagulant platelet creation. Therefore, this study aimed to elucidate whether lysosomal exocytosis and fibrinogen receptor activities of circulating normal-sized platelet subpopulations influence WB ɑ-thrombin-induced platelet activity in type 2 diabetes.

## Methods

### Ethics statements, patients, and blood sampling

After ethical approval (Regionala Etikprövningsnämnden, Medicinska Fakultetens Kansli, Linköpings Universitet, SE-581 83 Linköping (registration number 2018/54-31)) patients with type 2 diabetes (*n* = 16) were recruited (Table 1). The study adhered to the principles of Helsinki and written informed consent was obtained from all participants. A well-founded diagnosis of type 2 diabetes was the only inclusion criterion. There were no exclusion criteria. This disease was studied because it is a risk factor for thrombotic events. The patients joined a clinical control in primary care and were enrolled in 2020 when laboratory resources were available. Ethylenediamine tetra acetic acid (EDTA) anticoagulated WB (3 mL) was sampled for routine laboratory analysis (Table 1). For functional flow cytometry studies, WB (9 mL) was collected from the antecubital vein into 3.2% sodium citrate tubes and immediately transferred to an inhibitory cocktail [7,26,27] containing equal volumes of the following stock solutions:

1. 0.13 M of Na_2_EDTA and 0.15 M of Na_2_Citrate (pH 7.4 at 25 °C),
2. 2.7 mM of theophylline dissolved in 150 mM of TRIS chloride buffer (pH 7.4 at 25 °C), and
3. 1 mg/L of prostaglandin E_1_ dissolved in 95% (w/v) ethanol.

**Table 1.** Clinical and demographic characteristics of the patients with type 2 diabetes (*n* = 16).

Male/female (*n*)	9/7
Age (years)	70 ± 11 (SD)
Body weight (kg)	84 ± 11 (SD)
Duration of diabetes (years)	10 ± 5 (SD)
Previous myocardial disease (%)	25
Previous cerebral disease (%)	12
Insulin (%)	33
Metformin (%)	73
Other oral antidiabetic drugs (%)	40
β-Blockers (%)	53
Diuretics (%)	47
Ca^2+^-blockers (%)	53
Aspirin (%)	40
ADP receptor-blockers (%)	7
ACE or A2-inhibitors (%)	53
Statins (%)	75
Platelet count (×10^9^/L)	242 ± 64 (SD)
Neutrophil count (×10^9^/L)	4.1 ± 0.8 (SD)
Hemoglobin A1_c_ level (mmol/L)	57 ± 15 (SD)
Creatinine level (μmol/L)	78 ± 25 (SD)

SD: standard deviation; Ca^2+^: calcium; ADP: adenosine diphosphate; ACE: angiotensin-converting enzyme.

Then, this mixture was used for platelet density separation [7,26,27]. Platelet fractionation and flow cytometer studies commenced approximately 120 min after sampling.

### Platelet density fractionation

Previous studies have described how platelet density varies between 1.090 and 1.040 kg/L [10–12]. Thus, linear polyvinylpyrrolidone-coated silica gradients covering that span were used to separate the platelets [7]. To avoid activation in the test tubes, the gradients contained EDTA, prostaglandin E_1_, and theophylline. After centrifugation, the platelet population (i.e. the gradient) was split by gravidity into density subpopulations (*n* = 16) [7]. Thus, high-density corpuscles were in low-digit populations, so higher numbers denoted less-dense platelets.

### Flow cytometry

A Gallios flow cytometer (Beckman Coulter, Brea, CA) equipped with a multicolor design and three lasers (405, 488, and 638 nm) was used. The following platelet features were determined: (a) platelet size, normal-sized platelets, small platelets, and vesicles; (b) lysosomal exocytosis, surface-attached LAMP-1; (c) fibrinogen receptor (α_IIb_β_3_) activity, surface αIIbβ3 activation specific antibody (PAC-1); (d) platelet surface phosphatidylserine surface-bound annexin V; and (e) mitochondrial transmembrane potentials, retention of 1,1′,3,3,3′,3′-hexamethyl-indodicarbo-cyanine iodide (DiIC_1_(5)).

**Figure 1.**
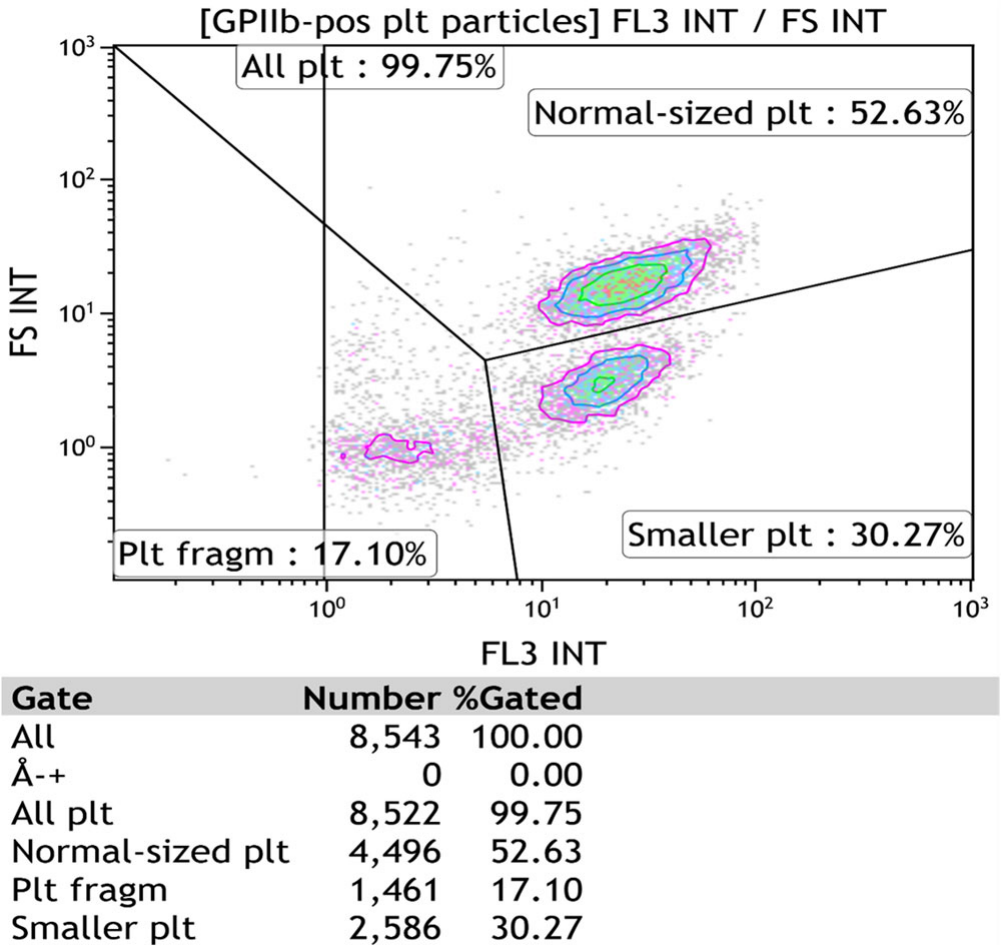
Shows for patient no. 16 whole blood size-dependent platelet populations after ɑ-thrombin (10 U/mL) provocation as determined by flow-cytometry. The illustration demonstrates the size-dependent platelet subfractions (normal-sized, small platelets, and vesicles) together with manual the gating of the device.

A flow cytometry protocol, first described by Ramström’s research group, was used without substantial alterations [4]. The antibodies and probes used are listed in Table 2. Platelets were recognized by forward scatter (size) and GPIIb receptor fluorescence, and normal-sized populations, as determined by flow cytometry gating, were studied. The probes and antibodies were added to the density subpopulations (Table 2) as described previously [4,26] and we did not extract platelets from the polyvinylpyrrolidone-coated silica. Gating has been extensively explained elsewhere [26] but is also shown in Figure 1. It demonstrates an individual sample after α-thrombin (10 U/mL) provocation and shows the gating of the GPIIb positive populations (normal-sized platelets, small platelets, and vesicles). In all samples, the proportions of positive corpuscles (%) were evaluated, and mean fluorescence intensities were not determined. The management of different controls has been described in detail [4]. After 10 min at room temperature the reactions were stopped by dilution in HEPES-Ca^2+^. Flow cytometry particle acquisition ended either after counting >5000 corpuscles or after 2 min. Thus, the number of assessed particles differed depending on the subpopulation corpuscle count.

**Table 2.** The flow cytometry technique, i.e. the determinations, antibodies, probes, final concentrations, manufacturers, and lot numbers.

Platelet identification Detection of the platelet receptor α_Iib_	ANTIBODY^a^ CD41; PE 0.69 μg/mL Lot number: 7628032
Lysosomal exocytosis Analysis of membrane-bound LAMP-1	ANTIBODY^a^ LAMP-1 (CD107a, clone: H4A39); PE 0.5 μg/mL Lot number: 9024681
Fibrinogen α_IIb_β_3_ receptor activity Analysis of membrane-bound PAC-1	PROBE^b^ PAC-1; FITC 0.56 μg/mL Lot number: 7093931
Determination of surface attached annexin V Membrane exposed phosphatidylserine	PROBE^b^ Annexin V-V450; PE 2.67 ng/mL Lot number: 0020903
Mitochondria membrane potential Determination of DiIC_1_(5) retention	PROBE^c^ 1,1′,3,3,3′,3′-hexamethyl-indodicarbo – cyanineiodide 30 nM

DiIC_1_(5): 1,1′,3,3,3′,3′-hexamethylindodicarbocyanine (relates inversely with mitochondria damage); FITC: fluorescein isothiocyanate; LAMP-1: lysosomal associated membrane protein; PAC-1: the activated fibrinogen receptor; PE: phycoerythrin.

^a^Beckman Coulter (Brea, CA).

^b^Becton Dickinson (Franklin Lakes, NJ).

^c^Molecular Probes (Eugene, OR).

### Data assessment and analysis

**Figure 2.**
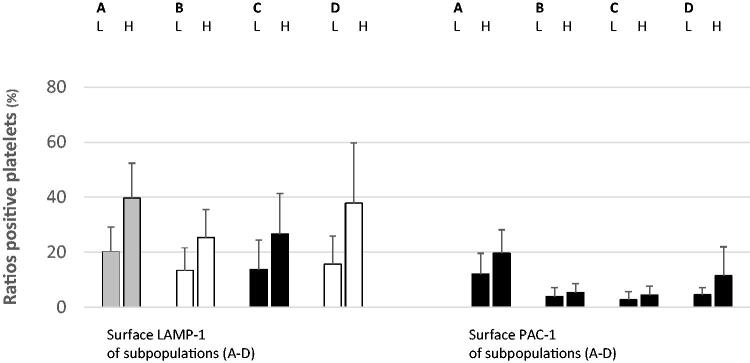
Surface LAMP-1 (left) and PAC-1 (right) expressions (mean ± SD) of circulating density-separated normal-sized platelets divided into low- and high-responders according to WB LAMP-1 expression after ɑ-thrombin provocation (10 U/mL). The colors indicate statistical significance (red, *p* < 0.01; yellow, *p* < 0.05; black, not significant) between the pairs. For each pair, the left bar summarizes WB ɑ-thrombin low-responders (L), and the right bar shows high-responding participants (H). A–D denote the density intervals. A: density subpopulations nos. 1–4; density span 1.090–1.079 kg/L. B: density subpopulations nos. 5–8; density span 1.079–1.067 kg/L. C: density subpopulations nos. 9–12; density span 1.067–1.054 kg/L. D: density subpopulations nos. 13–16; density span 1.054–1.040 kg/L. LAMP-1: lysosomal-associated membrane protein 1; nos.: numbers; PAC-1: fibrinogen receptor (α_IIb_β_3_) activity; SD: standard deviation; WB: whole blood. The number (mean ± SD) of normal-sized platelets evaluated using flow cytometry for the density fractions (nos. 1–16): (1) 87 ± 95; (2) 320 ± 311; (3) 594 ± 688; (4) 1244 ± 1812; (5) 2578 ± 1812; (6) 4631 ± 904; (7) 4814 ± 844; (8) 5138 ± 118; (9) 5261 ± 158; (10) 5305 ± 212; (11) 5139 ± 871; (12) 3831 ± 1738; (13) 2718 ± 1602; (14) 2053 ± 1813; (15) 1585 ± 1296; (16) 1012 ± 605.

**Figure 3.**
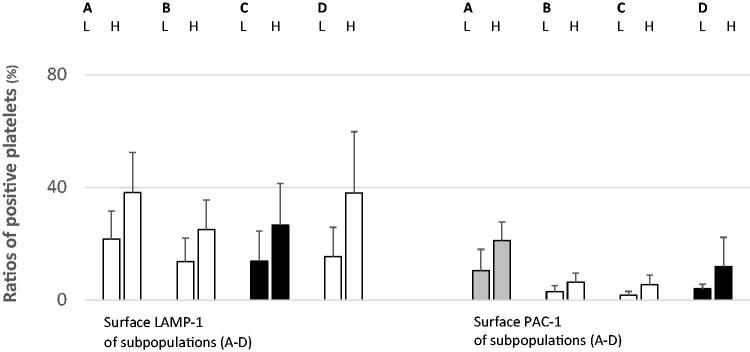
Membrane LAMP-1 (left) and PAC-1 (right) (mean ± SD) of density-separated normal-sized platelets split according to WB PAC-1 responses after ɑ-thrombin provocation (10 U/mL). The colors denote significance (red, *p* < 0.01; yellow, *p* < 0.05; black, not significant). For each pair, the left and right bars display WB ɑ-thrombin low- (L) and high- (H) responders, respectively. A–D denote the density intervals. A: density subpopulations nos. 1–4; density span 1.090–1.079 kg/L. B: density subpopulations nos. 5–8; density span 1.079–1.067 kg/L. C: density subpopulations nos. 9–12; density span 1.067–1.054 kg/L. D: density subpopulations nos. 13–16; density span 1.054–1.040 kg/L. LAMP-1: lysosomal-associated membrane protein 1; nos.: numbers; PAC-1: fibrinogen receptor (α_IIb_β_3_) activity; SD: standard deviation; WB: whole blood. The quantities (mean ± SD) of normal-sized platelets assessed by the flow cytometer apparatus for the density fractions (nos. 1–16) are given in the legend of Figure 2.

**Figure 4.**
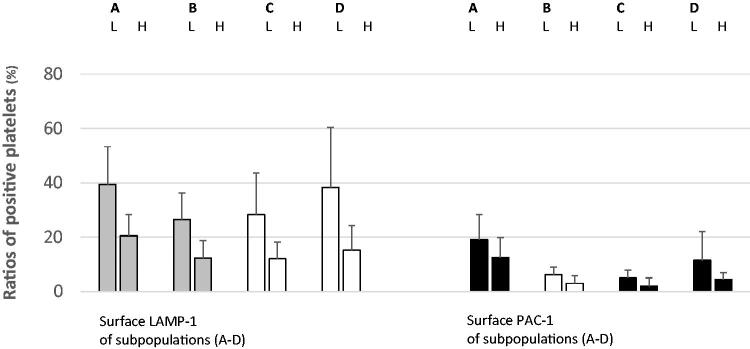
Surface LAMP-1 (left) and PAC-1 (right) (mean ± SD) expressions of density-separated ordinary platelets divided according to WB annexin V after ɑ-thrombin stimulation (10 U/mL). The colors illustrate statistical significance (red, *p* < 0.01; yellow, *p* < 0.05; black, not significant). For each couple, the left bar demonstrates WB ɑ-thrombin low-responders (L), and the right one shows their high-responding counterparts (H). A–D indicate the density intervals. A: density subpopulations nos. 1–4; density span 1.090–1.079 kg/L. B: density subpopulations nos. 5–8; density span 1.079–1.067 kg/L. C: density subpopulations nos. 9–12; density span 1.067–1.054 kg/L. D: density subpopulations nos. 13–16; density span 1.054–1.040 kg/L. LAMP-1: lysosomal-associated membrane protein 1; nos.: numbers; PAC-1: fibrinogen receptor (α_IIb_β_3_) activity; SD: standard deviation; WB, whole blood. The numbers (mean ± SD) of normal-sized corpuscles evaluated by the flow cytometer for the density fractions (nos. 1-16) are shown in Figure 2.

**Figure 5.**
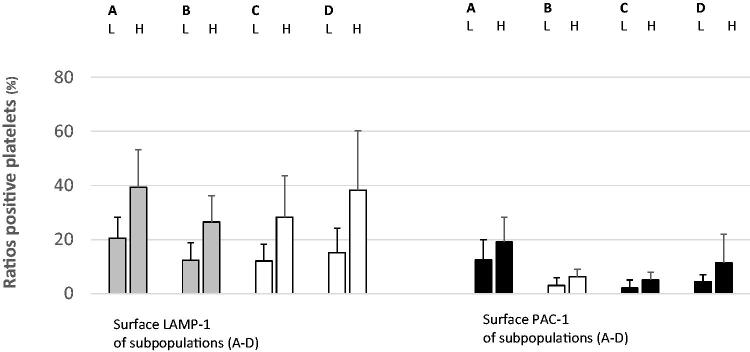
Surface LAMP-1 (left) and PAC-1 (right) (mean ± SD) of density separated normal-sized platelets divided according to WB DiIC_1_(5) responses after ɑ-thrombin provocation (10 U/mL). In this setting, a high DiIC_1_(5) denotes less disintegrated mitochondria. The colors designate the levels of significance (red, *p* < 0.01; yellow, *p* < 0.05; black, not significant). For each pair, the left bar (L) summarizes patients with more disintegrated WB platelet mitochondria after ɑ-thrombin provocation. The right bar (H) displays patients showing more retained organelles after stimulation. A–D denote the density intervals. A: density subpopulations nos. 1–4; density span 1.090–1.079 kg/L. B: density subpopulations nos. 5–8; density span 1.079–1.067 kg/L. C: density subpopulations nos. 9–12; density span 1.067–1.054 kg/L. D: density subpopulations nos. 13–16; density span 1.054–1.040 kg/L. DiIC_1_(5): mitochondrial transmembrane potential, that is, retention of 1,1′,3,3,3′,3′-hexamethylindodicarbocyanine iodide; LAMP-1: lysosomal-associated membrane protein 1; nos.: numbers; PAC-1: fibrinogen receptor (α_IIb_β_3_) activity; SD: standard deviation; WB: whole blood. The numbers (mean ± SD) of normal-sized corpuscles evaluated by the flow cytometer for the density fractions (nos. 1–16) are shown in Figure 2.

During the assessment, surface LAMP-1 was used as a sign of platelet lysosomal release, and PAC-1 indicated aggregatory corpuscles. Membrane-attached annexin V and mitochondrial damage identified procoagulant populations [4,26]. The flow cytometer recognized normal-sized platelets in density subpopulations (*n* = 16) [4,26]. For all patients (*n* = 16) and in all fractions (*n* = 16), the ratios (%) of normal-sized corpuscles that were positive for LAMP-1 were analyzed. This procedure was repeated for the activated α_IIb_β_3_ receptor (PAC-1). Subsequently, WB activity markers LAMP-1 (Figure 2), PAC-1 (Figure 3), annexin V (Figure 4), and DiILC_1_(5) (Figure 5) were determined after α-thrombin (10 U/mL) provocation in a separate sample. For all participants (*n* = 16), means and standard deviations of the ratio (%) (LAMP-1 or PAC-1) of neighboring (*n* = 4) density populations were calculated. Then, means were arranged into groups (*n* = 2) consisting of patients (each *n* = 8) with lower and higher *in vitro* ɑ-thrombin-induced WB activity as aforementioned. The unpaired Student *t*-test (Excel, Microsoft, Redmond, WA,) was used to perform statistical analysis. A *p*-value <0.05 was considered statistically significant.

## Results

Figure 2 shows that LAMP-1 expression of very dense platelets (A: subpopulations numbers nos. 1–4; density span 1.090–1.079 kg/L) (*p* < 0.01), semi-dense populations (B: subpopulations nos. 5–8; density span 1.079–1.067 kg/L) (*p* < 0.05), and light platelets (D: subpopulations nos. 13–16; density span 1.054–1.040 kg/L) (*p* < 0.05) was closely related to WB ɑ-thrombin-evoked (10 U/mL) lysosomal release (LAMP-1). In contrast, surface PAC-1 of density-separated circulating platelets did not affect WB LAMP-1 expression after provocation (Figure 2).

The ratio (%) of normal-sized platelets expressing LAMP-1 in the density subpopulations and WB ɑ-thrombin-evoked (10 U/mL) fibrinogen receptor activity were closely related (Figure 3). For subpopulations (A, B: nos. 1–8; density span 1.090–1.067 kg/L) and (D: subpopulations nos. 13–16; density span 1.054–1.040 kg/L), the levels of significance were <0.05. Figure 3 also shows the close relationship between normal-sized platelets expressing the activated fibrinogen receptor (PAC-1) and WB agonist-evoked α_IIb_β_3_ receptor activity. The *p*-values reached <0.01 for very dense populations (A: subpopulations nos. 1–4; density span 1.090–1.079 kg/L) and the cohorts (B–C: subpopulations nos. 5–12; density span 1.079–1.054 kg/L) displayed corresponding higher *p*-values (*p* < 0.05).

Figure 4 shows the proportions (%) of LAMP-1-expressing density-separated normal-sized platelets and inverse relationships with ɑ-thrombin-evoked (10 U/mL) WB annexin V. For A, B: subpopulations nos. 1–8 (density span 1.090–1.067 kg/L), the levels of significance were <0.01. Lighter platelets (C, D: subpopulations nos. 9–16; density span 1.067–1.040 kg/L) showed lower but still significant differences (*p* < 0.05). It is also apparent that the percentage of PAC-1-positive semi-dense platelets (B: subpopulation: nos. 5–8; density span 1.079–1.067 kg/L) and WB ɑ-thrombin-evoked annexin V were inversely associated (*p* < 0.05).

Figure 5 compares the ratios (%) of density-separated LAMP-1 positive platelets and WB ɑ-thrombin-induced (10 U/mL) DilC1(5). For A, B: subpopulations nos. 1–8 (density span 1.090–1.067 kg/L), the levels of significance were <0.01; the lower subpopulation surface LAMP-1 was associated with more disintegrated mitochondria after WB provocation. The corresponding *p*-values for lighter, normal-sized platelets (C, D: subpopulations nos. 9–16; density span 1.067–1.040 kg/L) were <0.05. The ratio (%) of PAC-1 expressing, medium dense normal-sized platelets (B: subpopulation nos. 5–8; density span 1.079–1.067 kg/L) also showed an inverse relationship with WB mitochondrial integrity after ɑ-thrombin provocation (*p* < 0.05) (Figure 4).

Platelet lysosomal release (LAMP-1) was lower in unstimulated WB than in semi-dense normal-sized subpopulations, indicating that centrifugation causes lysosomal release. In contrast, sample processing did not provoke the α_IIb_β_3_ receptor (PAC-1), surface annexin V, or mitochondrial disintegration (Table 3).

**Table 3.** Spontaneous whole blood lysosomal exocytosis (surface LAMP-1), fibrinogen receptor activity (PAC-1), annexin V, DiIC_1_(5), and corresponding surface activity markers of medium-dense normal-sized platelets subpopulations after processing and density separation.

	LAMP-1 (% of positive platelets)	PAC-1 (% of positive platelets)	Annexin V (% of positive platelets)	DiIC_1_(5) (% of positive platelets)
Whole blood^a^	4 ± 4	4 ± 3	2 ± 1	99 ± 1
Density subpopulation B^a,b^	19 ± 11	5 ± 3	2 ± 1	99 ± 1
Density subpopulation C^a,c^	20 ± 15	4 ± 3	3 ± 4	99 ± 2

A higher DiIC_1_(5) level suggests less mitochondria damage.

DiIC_1_(5): mitochondrial transmembrane potential, that is, retention of 1,1′,3,3,3′,3′-hexamethylindodicarbocyanine iodide; LAMP-1: lysosomal-associated membrane protein; nos.: numbers; PAC-1: the activated fibrinogen receptor.

^a^Mean ± standard deviation.

^b^B: density subpopulations nos. 5–8; density span 1.079–1.067 kg/L.

^c^C: density subpopulations nos. 9–12; density span 1.067–1.054 kg/L.

## Discussion

This descriptive study showed that platelet activity, that is, proportions of LAMP-1 expressing circulating normal-sized populations, although with some exceptions, are closely related to WB *in vitro* ɑ-thrombin-evoked (10 U/mL) platelet lysosomal release (LAMP-1) (Figure 2). Proportions were also associated with WB α-thrombin-induced fibrinogen receptor activity (PAC-1) (Figure 3) and inversely associated with procoagulant platelet reactions (i.e. surface annexin V (Figure 4) and mitochondrial injury (Figure 5) after provocation. It is to hypothesize that such previously unknown mechanisms affecting platelet reactivity offer new options for influencing platelets in type 2 diabetes.

Lysosomal exocytosis (surface LAMP-1) of normal-sized, semi-light platelets (C: subpopulations nos. 9–12; density span 1.067–1.054 kg/L) was not associated with ɑ-thrombin-provoked LAMP-1 and the α_IIb_β_3_ receptor PAC-1 activity (Figures 2 and 3). This finding suggests that platelet heterogeneity affects ɑ-thrombin-induced lysosomal release and fibrinogen receptor activity *in vitro*. The percentage of circulating normal-sized platelets displaying activated fibrinogen receptors promoted ɑ-thrombin-evoked α_IIb_β_3_ reactions, i.e. aggregation (Figure 3), but failed to modify the WB lysosomal discharge (Figure 2). Such platelets, although to a lesser degree, affected the generation of procoagulant platelets (Figures 4 and 5).

To avoid platelet activation during density separation, citrate-anticoagulated WB was added instantaneously to an inhibitory mixture containing EDTA and prostaglandin E_1_. The gradients also contained such compounds [7,26,27]. Sample handling neither activated α_IIb_β_3_ receptors nor the creation of procoagulant platelets. In contrast, the surface LAMP-1 levels were higher after laboratory processing (Table 3). The blocking solution also makes it impossible to use subpopulations for functional studies.

For normal-sized platelets, statistical evaluation was performed for the mean values of multiple density intervals (*n* = 4). LAMP-1 expression in these platelets was closely related to ɑ-thrombin-induced (10 U/mL) lysosomal release and αIIbβ3 receptor activation (Figure 2). The ratios of LAMP-1 expressing normal-sized density subpopulations were also inversely linked to procoagulant platelet creation after agonist provocation (Figures 4 and 5). It is possible that such multiple associations make statistical errors unlikely. Subpopulations showed substantial individual variations with respect to surface LAMP-1 (Figures 2–5), but WB platelet counts (Table 1) and platelet activity in response to ɑ-thrombin also displayed large standard deviations. Consequently, heterogeneity at the baseline may partly explain the individual divergence after sample processing.

Community-dwelling seniors who visited an outpatient clinic for type 2 diabetes were enrolled. We wanted to study a procoagulant condition with respect to differences within the diseased group so we did not include healthy individuals as controls; therefore, our findings could be features of disease or they may occur in health as well. The participants were heterogeneous with respect to comorbidities and medications (Table 1), which may have influenced the results. As judged from the medications (Table 1), elevated cholesterol and hypertension were common. When providing consent, some patients were taking either ADP receptor blockers (*n* = 1) or aspirin (*n* = 6). Platelet ɑ-thrombin responses were unrelated to aspirin (data not shown), but clopidogrel affects agonist-evoked platelet WB activity [28].

The current study investigates normal-sized platelets as defined by flow cytometry gating (Figure 1) [26]. This made it possible to study a homogeneous population with respect to size, as the device excluded small platelets and extracellular vesicles. Flow cytometry did not quantify the individual platelets with respect to the degree of activation. It is thus possible that assessment of the fluorescence strength of activated platelets would provide a better understanding of platelet reactions. Type 2 diabetes is characterized by an increased mean platelet volume but in this study WB platelet size was measured in EDTA anticoagulated blood by an automatic cell counter. The time between sampling and analysis was not standardized. Thus, we did not evaluate WB mean platelet volumes scientifically.

This study verifies findings of previous work [26,27] by showing that circulating normal-sized high-density (A: subpopulations nos. 1–4; density span 1.090–1.079 kg/L) and low-density (D: subpopulations nos. 12–16; density span 1.054–1.040 kg/L) platelets display more enhanced α_IIb_β_3_ activity *in vivo* than intermediate-density cohorts (Figure 2). This work broadens the observations by demonstrating that surface LAMP-1 (Figure 2) and annexin V (Figure 4) of resting normal-sized platelets behaved similarly with high- and low-density subpopulations, showing increased *in vivo* activities.

Possible platelet agonist responses involve lysosomal release, enhanced fibrinogen receptor activity, and an increased procoagulant platelet count. This study did not explain the mechanisms that bifurcate WB platelets in performing different tasks when activated. It is not to confuse association with causality, as the latter requires more in-depth experimentation also including animal studies. It makes it obligatory to appraise the practical consequences of the current findings in forthcoming research. However, in type 2 diabetes, lysosomal release, as estimated from the surface LAMP-1 of circulating ordinary platelets, forecasts *in vitro* WB reactions (lysosomal discharge and α_IIb_β_3_ receptor activity) after ɑ-thrombin provocation (10 U/mL) (Figures 2 and 3). The marker also showed inverse relationships with agonist-induced procoagulant platelet formation (Figures 4 and 5). Thus, lysosomal discharge of circulating normal-sized platelets associated with ɑ-thrombin-induced WB platelet response *in vitro*.

## Conclusions

Lysosomal exocytosis of circulating density-separated normal-sized platelets forecasts *in vitro* WB ɑ-thrombin-induced (10 U/mL) responses in type 2 diabetes. Higher proportions of circulating platelets expressing LAMP-1 promoted WB lysosomal release and α_IIb_β_3_ receptor activity, and lower ratios enhanced WB procoagulant platelet production. It is to hypothesize that the new described regulatory mechanism offers new ways to influence platelet behavior in type 2 diabetes.

## Data Availability

Raw data are available from the corresponding author upon reasonable request.
